# RNA-binding proteins as a common ground for neurodegeneration and inflammation in amyotrophic lateral sclerosis and multiple sclerosis

**DOI:** 10.3389/fnmol.2023.1193636

**Published:** 2023-07-04

**Authors:** Isabel Acosta-Galeana, Ricardo Hernández-Martínez, Tania Reyes-Cruz, Erwin Chiquete, Jose de Jesus Aceves-Buendia

**Affiliations:** ^1^Facultad de Química, Universidad Nacional Autónoma de México, Mexico City, Mexico; ^2^Facultad de Ciencias Químicas, Universidad Veracruzana, Xalapa, Mexico; ^3^Laboratorio de Biología Molecular, División de Ciencias Biológicas y de la Salud, Universidad Autónoma Metropolitana, Mexico City, Mexico; ^4^Departamento de Neurología y Psiquiatría, Instituto Nacional de Ciencias Médicas y Nutrición Salvador Zubirán, Mexico City, Mexico

**Keywords:** TDP-43, ALS, MS, neurodegeneration, DNA-binding proteins, RNA-binding proteins, autoimmunity

## Abstract

The neurodegenerative and inflammatory illnesses of amyotrophic lateral sclerosis and multiple sclerosis were once thought to be completely distinct entities that did not share any remarkable features, but new research is beginning to reveal more information about their similarities and differences. Here, we review some of the pathophysiological features of both diseases and their experimental models: RNA-binding proteins, energy balance, protein transportation, and protein degradation at the molecular level. We make a thorough analysis on TDP-43 and hnRNP A1 dysfunction, as a possible common ground in both pathologies, establishing a potential link between neurodegeneration and pathological immunity. Furthermore, we highlight the putative variations that diverge from a common ground in an atemporal course that proposes three phases for all relevant molecular events.

**Graphical abstract:**
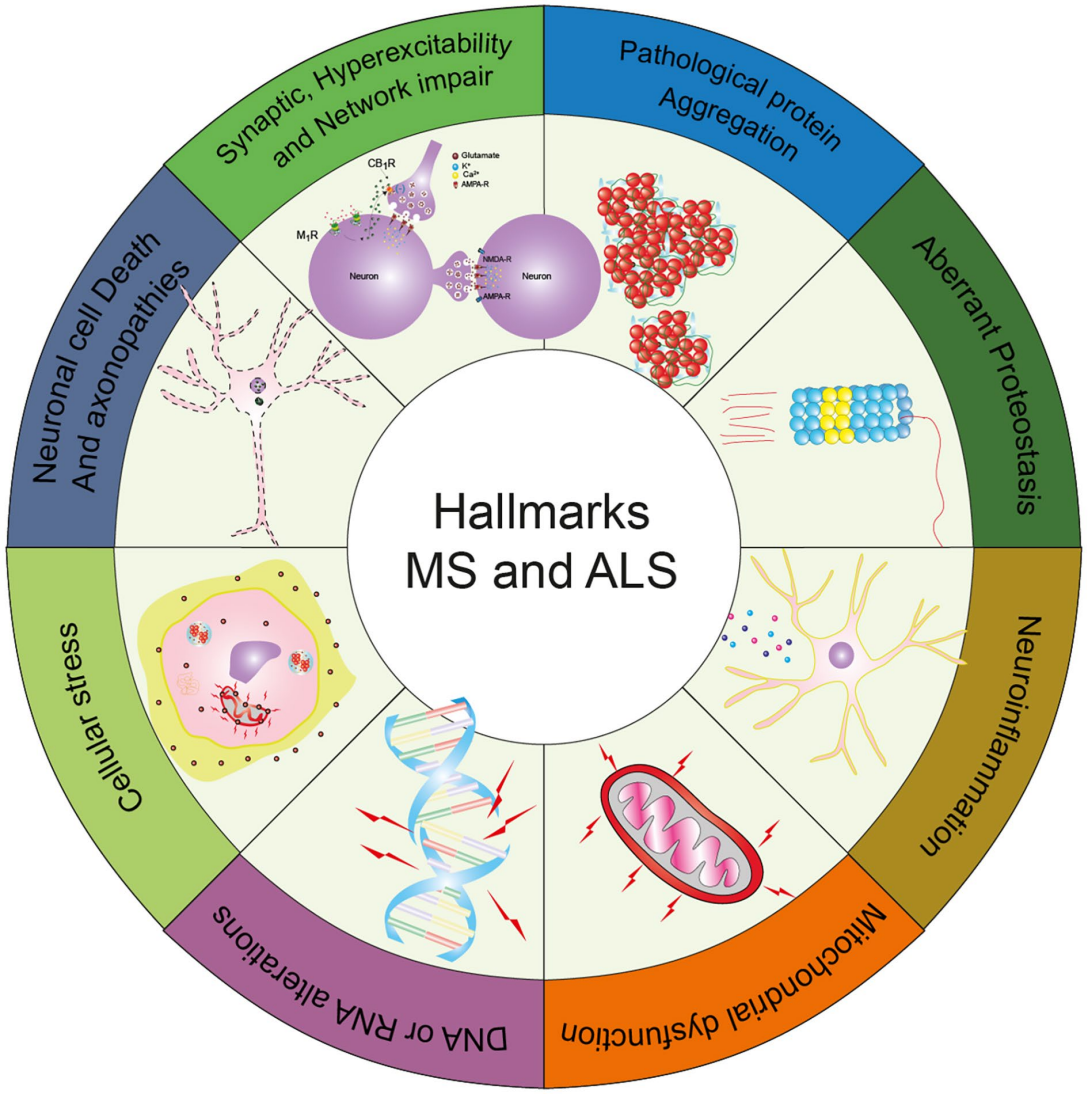
The graphical abstract displays the common hallmarks between Amyotrophic Lateral Sclerosis and Multiple Sclerosis, as described in this review [adapted from Wilson et al. (2023)].

## 1. Introduction

Neurodegenerative diseases can affect the nervous system in a wide range of manners, affecting cognition in the case of frontotemporal dementia (FTD) to independent movement in the cases of amyotrophic lateral sclerosis (ALS) and multiple sclerosis (MS). Even though these entities seem to be very different from the clinical standpoint, at the molecular level these disorders share some characteristics such as protein aggregation, metabolic dysfunction, abnormalities in the regulation of proteostasis, neuroinflammation, synapse malfunction, neuronal network disruption, and neurotoxicity, among others features (Wilson et al., 2023).

ALS is considered a neurodegenerative condition with a very poor prognosis. The main characteristics observed in this condition are the death of motor neurons (MNs) and conglomerates of ribonucleic acid binding proteins (RBPs). These hallmarks result in permanent and irreversible loss of mobility, with a fatal prognosis (Feldman et al., 2022).

A growing number of molecular components associated with ALS have been identified, which includes the formation of protein aggregates; such as fused in sarcoma protein (FUS), deoxyribonucleic acid binding proteins such as TAR DNA-binding protein 43 (TDP-43), optineurin (OPTN), and the presence of a hexanucleotide repeat expansion in the *C9orf72* gene or mutations of the superoxide dismutase 1 (SOD1) gene (Berdyński et al., 2022; Suzuki et al., 2023).

The anatomical areas involved in the pathology of ALS include the central nervous system (CNS), spinal cord, and some extra motor areas, where the implication of the immune system is observed, leading to permeabilization of the blood–brain barrier (BBB), activation of microglia and increase of T lymphocytes within the CNS (Holmoy, 2008). This permeabilization of the CNS is responsible for the infiltration of proinflammatory cytokines, causing tissue damage and exacerbating the disease, hence the lethal prognosis for ALS (Brown and Al-Chalabi, 2017; Liu T. et al., 2017). Although there are several clinical stages, core features include muscle weakness and atrophy, fasciculations, and brisk tendon reflexes (Van Es et al., 2017).

MS is another neurodegenerative disease whose main characteristic is demyelinating damage in neuronal axons, caused by the activation of the innate and adaptive immune response ultimately leading to CNS degeneration (Axisa and Hafler, 2016). MS is traditionally described as an inflammatory non-proteinopathy neurodegenerative disorder, but a growing body of evidence suggests that abnormal protein aggregation occurs in human oligodendrocytes (OLs; Rohan et al., 2014; Masaki et al., 2020).

The infiltration of T and B lymphocytes to the CNS can only occur after the activation of the innate immune response. This phenomenon is mediated through the recognition of damage-associated molecular patterns (DAMPs) by pathogen recognition receptors (PRRs). This signaling pathway generates inflammation at a peripheral level, causing permeability of the BBB. Activated T and B lymphocytes produce proinflammatory cytokines that cause neuronal damage at different levels, particularly B lymphocytes. These lymphocytes produce specific antibodies against myelin (Maglione et al., 2021).

MS is more frequent in women than in men and in the young ages of 20–40 years than in other age groups. A few environmental risk factors have been identified so far, such as pre-infection by the Epstein–Barr virus (EBV), vitamin D deficiency, and latitude of residence (Garg and Smith, 2015). MS is a spectrum disorder, including clinical entities ranging from clinically isolated syndrome (CIS), relapsing–remitting MS (RRMS), primary progressive MS (PPMS), and secondary progressive MS (SPMS; Klineova and Lublin, 2018). A more recent approach suggests that MS should be looked at as a continuum of different syndromes with or without evidence of disease activity (Kuhlmann et al., 2023).

ALS and MS, although different entities in nature, clinical presentation, and disease progression show several pathological similarities, which are summarized in Table 1.

**Table 1:** Molecular characteristics of degeneration and inflammation in MS, ALS, EAE, and some ALS models.

Characteristic	ALS	ALS models	MS	EAE
Mislocalization of RBPs	Cook et al. (2020), Gruijs da Silva et al. (2022), Paul et al. (2021), Ryan et al. (2022)	Kim et al. (2021)Liu et al. (2016), Wegorzewska et al. (2009)	Salapa et al. (2018), Masaki et al. (2020)	Salapa H. A.-O. et al., 2020, Salapa H. E. et al., 2020
TDP-43 aggregates			Salapa et al. (2018), Salapa H. A.-O. et al. (2020), Salapa H. E. et al. (2020)	Salapa H. A.-O. et al., 2020, Salapa H. E. et al., 2020
TDP-43 dysfunction in oligodendrocytes	Rohan et al. (2014), Pons et al. (2020), Valori and Neumann (2021)	Lu et al. (2016)	Masaki et al. (2020)	N/A
hnRNP A1 aggregates	Kim et al. (2013), Purice and Taylor (2018)	N/A	Clarke et al. (2021), Salapa et al. (2018), Salapa H. A.-O. et al. (2020), Salapa H. E. et al. (2020), Jahan-Abad et al. (2023)	Salapa H. A.-O. et al., 2020, Salapa H. E. et al., 2020
Mitochondrial dysfunction	Wang et al. (2016), Dafinca et al. (2021), Gautam et al. (2022)	Altanbyek et al. (2016)	Barcelos et al. (2019), Blagov et al. (2022)	Nikić et al., 2011
Damage in Mitochondria structure and mtDNA	Wang et al. (2016), Zuo et al. 2021, Gautam et al. (2019)	Wang et al. (2016)	Barcelos et al. (2019), Blagov et al. (2022)	N/A
Stress Granules formation	Kirkinezos et al. (2005), Li et al. (2013), Berdyński et al. (2022), Ratti et al. (2020)	N/A	Douglas et al. (2016), Clarke et al. (2021), Libner et al. (2020)	N/A
Dysregulation of Autophagy	Rudnick et al. (2017), Evans and Holzbaur (2019)	Paul et al. (2023)	N/A	N/A
Activation of innate immune response against PPRs	Bright et al. (2021)	N/A	Mcginley et al. (2018), Lopes Pinheiro et al. (2016)	N/A
Permeabilization of BBB	Holmoy (2008), Beers et al. (2017)	N/A	Mcginley et al. (2018); Sospedra and Martin (2016)	N/A
Increased expression of IL-1b and TNF	Bright et al. (2021), Guidotti et al. (2021), Hu et al. (2017)	N/A	Beers et al. (2017), Rossi et al. (2014)	N/A
Induction Th1 and Th17 cells in CNS	N/A	N/A	Mcginley et al. (2018); Nitsch et al. (2021), Josefowicz et al. (2012)	N/A
Activation lymphocyte B and autoimmunity	N/A	N/A	Josefowicz et al. (2012), Ransohoff (2018)	N/A
Hyperexcitability	Hossaini et al. (2011), Perkins et al. (2021), Almad et al. (2022)	N/A	Rossi et al. (2014), Pitt et al. (2000), Senol et al. (2022)	N/A
Neurodegeneration in the spinal cord	Mey et al. (2023), Levin et al. (2014)	Liu et al. (2016)	Zeydan et al. (2018), Bischof et al. (2022)	Salapa H. A.-O. et al., 2020, Salapa H. E. et al., 2020
Antibodies against RBPs	Conti et al. (2021), Nielsen et al. (2021)	N/A	Yukitake et al. (2008), Lee et al. (2011)	Libner et al. (2020)
Disease severity/progression and RBPs aggregation	Brettschneider et al. (2013, 2014)	Philips and Rothstein (2015)	N/A	Salapa H. A.-O. et al. (2020), Salapa H. E. et al. (2020)
RBPs accumulation in intramuscular nerve bundles	Kurashige et al. (2022)	N/A	N/A	N/A

N/A, not available.

In this review, we look into some of the hallmarks of these two pathologies but make special emphasis on the pathological aggregation of proteins, specifically RBPs as a shared feature between both diseases.

## 2. Common features in pathophysiology

### 2.1. RBPs mislocalization by cellular stress

RBPs are a diverse set of proteins that play crucial roles in ribonucleic acid (RNA) splicing, transport, translation, and stability. The presence of these RBPs proteins is necessary to regulate proper cell function, being essential for the maintenance of cell homeostasis (Tesco and Lomoio, 2022). Its main characteristic is to regulate the expression of a great diversity of genes, which confers stability to messenger RNA (mRNA).

These RBPs have different binding domains to RNA and other proteins, which is why they are multifunctional proteins (Šušnjar et al., 2022). The absence or modification of one of these RBPs could cause a loss and/or gain of cellular function and result in a pathological state (Ash et al., 2010; Kraemer et al., 2010).

Some of these modifications occur within the nucleus, for example, the transcription of *C9orf72* produces transcripts that can form secondary RNA structures that have the ability to sequester RBPs, resulting in the formation of RNA foci (Wang Z. F. et al., 2019).

In ALS, the subsequent translation of the *C9orf72* gene results in the production of dipeptide repeat proteins (DPRs) that not only disrupt the nucleocytoplasmic transport machinery but also cause TDP-43 mislocalization Table 1 (Ryan et al., 2022).

**Figure 1:**
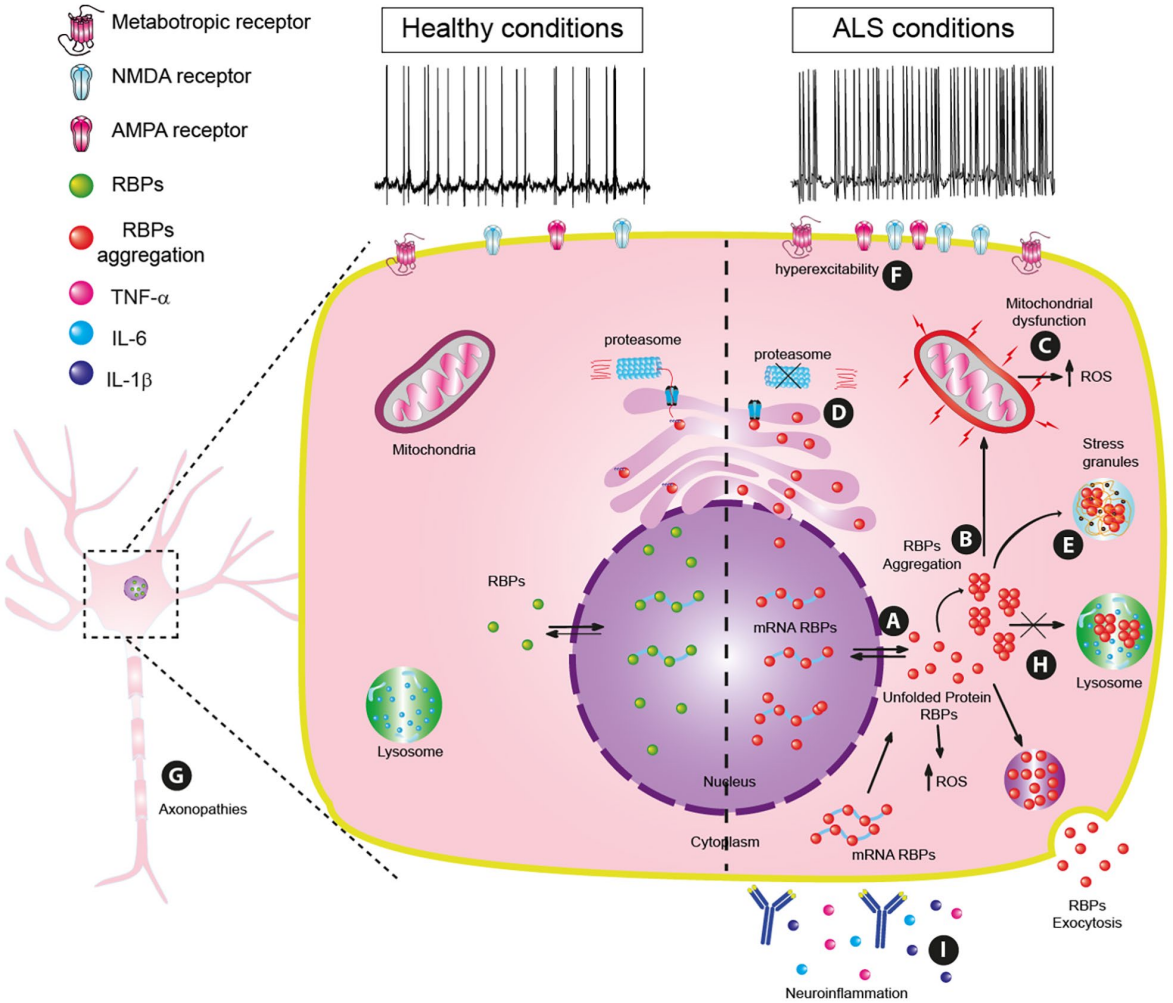
Principal pathophysiology mechanisms in amyotrophic lateral sclerosis (ALS). **(A)** RBP mislocalization by cellular stressors. **(B)** RBPs dysfunctionality: misfolding, and aggregation. **(C)** Mitochondrial dysfunction. (**D)** Proteostasis dysregulation. **(E)** Generation of stress granules. **(F)** Hyperexcitability. **(G)** Axonopathies. **(H)** Lysosome dysfunction and Autophagy Dysregulation. **(I)** Neuroinflammation.

These mislocalizations of RBPs might be triggered during disrupted proteostasis by heat shock, hyperosmolarity, oxidative stress (Chang et al., 2013; Sama et al., 2013; Chen et al., 2016; Hock et al., 2018) or by a mutated transportin, a nuclear import receptor (Dormann et al., 2010; Neumann et al., 2012). Additionally, mutations in the RBP genes and/or aberrant post-translational modifications may cause RBP to precipitate and aggregate (Bosco et al., 2010; Dewey et al., 2011; Figure 1A).

The cell will turn on protective mechanisms against aggregation, like TDP-43 phosphorylation in the C-terminal region (Gruijs da Silva et al., 2022) or control by chaperon proteins like the heat shock proteins HSP70, HSP90, and HSPB1 (Lin et al., 2021; Lu et al., 2022; Carrasco et al., 2023) in order to overcome aggregation but, if these mechanisms are unsuccessful, then the precipitation of aberrant aggregates will progress.

In the case of neurodegenerative diseases, there is growing evidence that shows that an excessive increase in reactive oxygen species (ROS) leads to cellular problems, including cell death (Patel, 2016; Wilson et al., 2023). A significant percentage of patients with ALS were identified with mutated SOD1 which is a very important defense enzyme against oxidative stress (Berdyński et al., 2022). These mutations modify the catalytic speed of SOD1, allowing the generation of ROS such as peroxynitrite, increasing the nitration of proteins (Pansarasa et al., 2018).

These mismanagements of oxidative stress and inefficient protective mechanisms will result in misfolding and mislocalization of nuclear RBPs such as FUS, TDP-43, or co-aggregates with poly (GR) dipeptides Table 1 (Kino et al., 2011; Patel, 2016; Cook et al., 2020; Ratti et al., 2020). TDP-43 actively participates in the generation of cytoplasmic inclusions such as P-Bodies and stress granules (SGs; Li et al., 2013; Guo et al., 2018) and causes damage to different organelles including mitochondria (Islam, 2017).

Some mechanisms controlling gene expression in MS are post-transcriptional RNA modifications such as oxidation. In RNA, the nucleoside that is more susceptible to oxidation is guanosine, resulting in the mutagenic 8-oxoguanosine. This molecule not only binds to its natural pair but also gains the ability to bind to adenosine, leading to defective proteins.

In an MS oxidative microenvironment, mRNAs are not equally affected. This phenomenon may explain why some molecules such as N-acetyl-aspartate transferase 8 (NAT8L) mRNA are expressed at lower levels. Its cognate protein is involved in the catalytic synthesis of N-acetyl aspartic acid (NAA), required for myelin synthesis and its decrease is a pathological characteristic (Sumi et al., 2017; Kharel et al., 2023).

**Figure 2:**
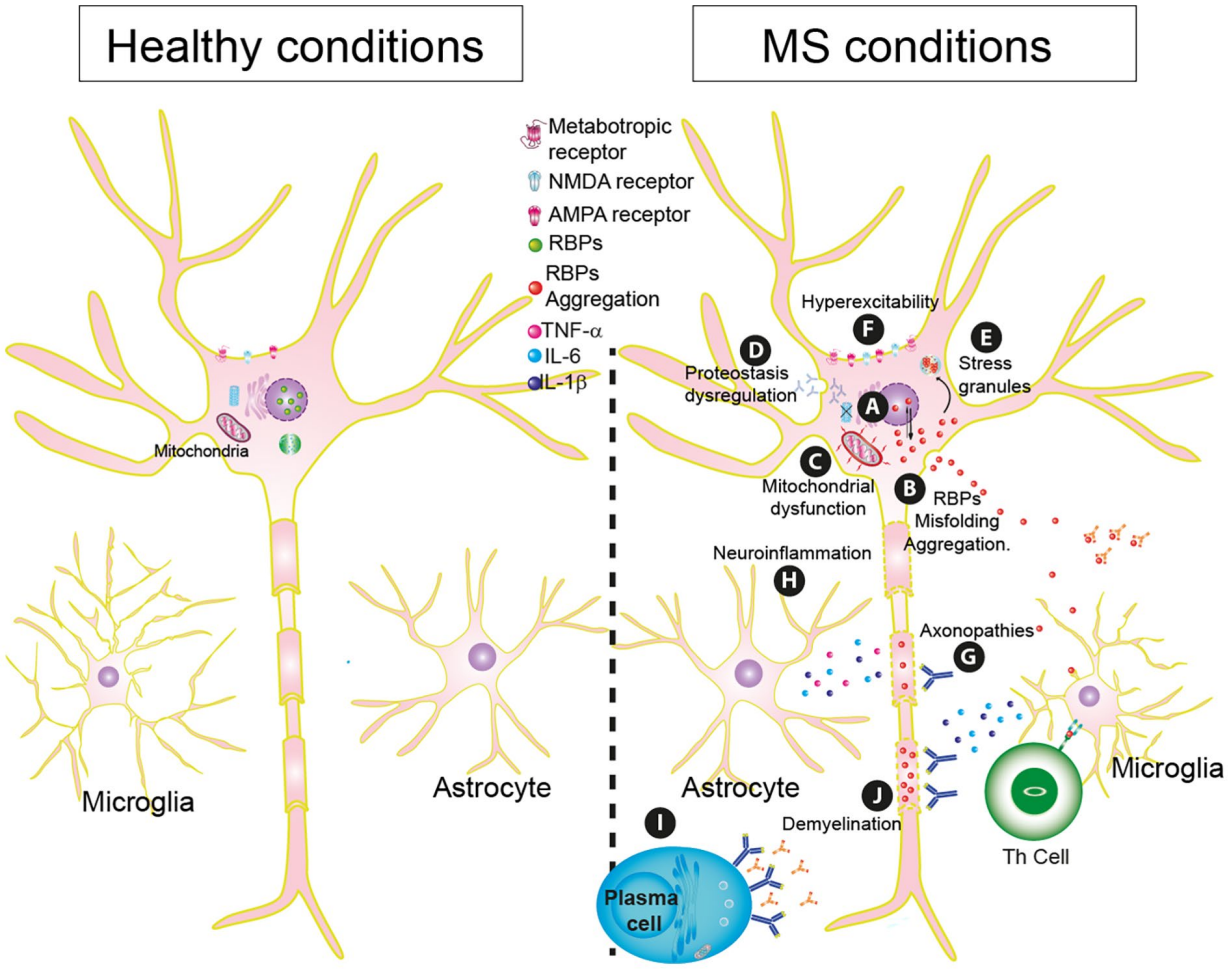
Essential pathophysiology mechanisms in Multiple sclerosis (MS). **(A)** RBP mislocalization by cellular stressors. **(B)** RBPs dysfunctionality: misfolding, and aggregation. **(C)** Mitochondrial dysfunction. **(D)** Proteostasis dysregulation. **(E)** Generation of stress granules. **(F)** Hyperexcitability. **(G)** Axonopathies. **(H)** Neuroinflammation. **(I)** Plasma Cells. **(J)** Demyelination.

Another consequence of oxidative stress in MS is the oxidation of phosphatidylcholine, one of the end-product markers of oxidative stress. It was found to be associated with injury and neuroinflammation (Qin et al., 2007). The presence of this produces neurodegeneration in the white matter of the spinal cord and demyelination (Dong et al., 2021; Figure 2A).

### 2.2. RBPs dysfunctionality: misfolding and aggregation

TDP-43, which is coded by the *TARDBP* gene, has been identified as a significant contributor to the development of ALS (Gao et al., 2019; Chhangani et al., 2021). Mutations in the *TDP-43* gene may favor the aggregation of misfolded TDP-43 proteins, achieving a prion-like configuration (Buratti, 2015). The anomalous accumulations of TDP-43 are commonly known as “TDP-43 proteinopathy” (Gao et al., 2019; Cook et al., 2020; Figure 1B; Table 1).

TDP-43 proteinopathy in ALS interferes with multiple pathways including RNA metabolism (Polymenidou and Cleveland, 2011), protein translation (Freibaum et al., 2010; Tollervey et al., 2011), stress-induced response (Colombrita et al., 2009), autophagy (Xia et al., 2016), endocytosis (Liu G. et al., 2017), ubiquitin-proteasome system and mitochondrial function (Wang et al., 2016).

Even though protein aggregation is not regarded as primary damage in MS pathology, increasing evidence suggests that the clustering of proteins like RBPs may have a crucial part in the progression of the condition, which could lead to both neurodegeneration and inflammation.

This RBP protein dysfunction has been observed in the experimental model of autoimmune encephalomyelitis (EAE) (Salapa H. A.-O. et al., 2020). In the spinal cord tissue, there is mislocalization and SGs formation of TDP-43 and heterogeneous nuclear ribonucleoprotein A1 (hnRNP A1). Interestingly, there is a positive correlation between EAE score and RBPs mislocalization. This correlation has been reported for ALS as well (Brettschneider et al., 2013, 2014; Kim et al., 2013; Philips and Rothstein, 2015; Purice and Taylor, 2018; Figure 2B). Knock-down of hnRNP A1 *in vitro* reduced neurite outgrowth, suggesting that the loss-of-function of this RBP by cytoplasmic mislocalization disrupts very important biological processes and leads to cell death (Anees et al., 2021).

To this day, there is no information available on whether RBPs can be found pathologically accumulated in the spinal cord gray matter of patients, but spinal cord atrophy is reported (Zeydan et al., 2018; Bischof et al., 2022). RBPs accumulation is reported for OLs; TDP-43 is mislocalized in active lesions in patients with MS. TDP-43 aggregates in OLs have also been described in ALS (Masaki et al., 2020; Valori and Neumann, 2021) but, in this pathology, OLs proliferation is increased, producing defective mature cells (Table 1). Normal physiological TDP-43 is needed for myelination and OLs survival; it was also found that RPB depletion in these cells does not affect MNs or the neuromuscular junction, suggesting that neurodegeneration could be an independent phenomenon that requires its own triggers (Wang et al., 2018).

Staufen1 protein (STAU1) is another RBP that forms SGs and has been recently linked to ALS pathology (Paul et al., 2021). STAU1, a mammalian target of rapamycin (mTOR) and phosphorylated-Ser2448-mTOR was found to be highly abundant in fibroblasts from ALS patients with reported TDP-43 mutations.

Nuclear accumulation of STAU1 has been described for an ALS/FT model in drosophila and it has been proposed as a possible new hallmark for the disease (Kim et al., 2021). It is reported that cell cultures with over-expressed STAU1 increased mTOR levels by mTOR-mRNA interaction which impaired autophagy (Paul et al., 2023).

#### 2.2.1. RNA metabolism

TDP-43 in ALS can negatively regulate the expression of various genes at different levels: from transcription, splicing to translation. TDP-43 interacts with proteins that regulate mRNA metabolism such as hnRNP, RNA helicases, splicing factors, translation regulatory proteins, and proteins that support and stabilize mRNA, also could be associated with transcription factors (Pons et al., 2017; Šušnjar et al., 2022).

TDP-43 is crucial for maintaining a variety of mRNAs, including those encoding synaptic proteins, choline acetyltransferase, FUS protein, and progranulin. These pre-mRNAs are directly linked to TDP-43 at various sites called long introns (Ishigaki and Sobue, 2018).

Moreover, TDP-43 acts as a regulator of itself to reduce its expression levels in the cell, it binds to the 3′ UTR region of its own pre-mRNA and prevents its maturation and subsequent translation, a mechanism that can be seen altered in ALS since the autoregulatory capacity of TDP-43 does not exist or it is diminished (Koehler et al., 2022).

Once the cytoplasmic aggregates of TD-P43 are formed and there is a decrease in the quantity of nuclear TDP-43, the splicing is decreased in the 3’ UTR portion, causing the amount of TDP-43 produced in the cytoplasm to rise. The mature TDP-43 mRNA that continues to replicate in the cytosol is an aberrant form in function that is only present in the cytoplasm. This causes the amount of TDP-43 aggregates to increase rapidly, disrupting TDP-43 autoregulation. This molecular process underlies the degeneration in ALS disease (Pons et al., 2017).

Small non-coding RNAs are 20–22 nucleotides in size and are regulatory factors for gene expression. In the particular case of ALS, TDP-43 can be associated with a microRNA (miRNA) formation complex (DGCR8) allowing its direct union with a nuclear complex called Drosha which is essential for the processing of immature RNAs (pri-miRNA). TDP-43, by facilitating the union of Drosha, produces mRNA, ready to enter its maturation process during transcription (Ha and Kim, 2014; Pham et al., 2020).

Cytoplasmic TDP-43 can also be associated with the Dicer complex (which contains TRBP). This interaction facilitates the processing of specific pre-miRNAs, which were known to be transported only in the nucleus, but it has now been established that there is a subset of pre-miRNAs (inmadure RNA) whose production is regulated by TDP-43 in the cytosol. This is a unique function of TDP-43 not only carried out in the nucleus but also in the cytoplasm (Polymenidou and Cleveland, 2011).

TDP-43 mislocalization due to accumulation in cytoplasmic aggregates may reduce the correct processing of miRNAs by TDP-43 and their binding to Drosha and Dicer. For example, a miRNA negatively regulated by the presence of TDP-43 aggregates is miR-132-3p, which is found abundantly in neurons and promotes neuronal growth by reducing levels of the GTPase-activating protein (Paez-Colasante et al., 2020; Pham et al., 2020).

Other miRNAs (miR-143-3p, −574-5p, and -133b) have been reported to be overexpressed during ALS disease, which correlated with the presence of TDP-43 clusters in the cytoplasm, which favored the metabolism of miRNAs by the Drosha and Dicer complex (Koehler et al., 2022). These results show that the up-expression or down-expression of certain miRNAs may serve as biomarkers of ALS severity.

In MS, as in ALS, there are reports of gene deregulation due to the presence of RBPs; some miRNAs bind to these proteins and modify their expression, degradation, or autoregulation at different levels. This suggests that these proteins have regulatory mechanisms through these miRNAs that may contribute to the pathogenesis of the disease (Consiglio et al., 2023).

Specifically, in MS, it has been reported that there is deregulation in the expression of some miRNAs. miR-29b is highly expressed in T lymphocytes (CD4^+^); This same miRNA (miR-29b) is down-expressed in activated T lymphocytes, which contributes to the pathogenesis of MS since it regulates the T helper response (Th1) of T lymphocytes causing chronic inflammation (Smith et al., 2012; Gao et al., 2021).

Another example of deregulation of gene expression is the case of miR-15a and miR-16-1, these are found at low levels of expression in mononuclear cells in the blood of patients with MS; Likewise, these miRNAs are found in T lymphocytes (CD4^+^) and also have effects on the B-cell lymphoma 2 gene (Bcl-2) in these lymphocytes, which delays apoptosis. In other words, the sustained presence of these miRNAs may help to maintain the chronic inflammation observed in MS (Lorenzi et al., 2012; Gao et al., 2021).

There is also dysregulation in miR-155; This is necessary for the proper functioning of the immune system as it controls and reduces the production of B cells in the lymph nodes. The presence of proinflammatory cytokines such as nuclear factor kappa-light-chain-enhancer of activated B cells (NF-κβ) induces the expression of miR-155 also in B and T lymphocytes, which contributes to perpetuating inflammation in the CNS in MS (Kamphuis et al., 2015; Chen et al., 2018).

In Th17 lymphocytes miR20b is decreased, while miR-21 and miR-590 are overexpressed. These miRNAs are directly involved in the differentiation of Th17 lymphocytes during MS disease, favoring their concentration in the CNS and with this the chronic activation of inflammation and autoimmunity (Murugaiyan et al., 2015).

miR-125a-2p was detected at a very high quantity in the cerebrospinal fluid (CSF) of patients with active demyelinated lesions. miR-125a-2p upregulation is also reported in brain samples from patients with white/gray matter active lesions, specifically. Data from mice suggests a potential role of miR-125a-2p in OLs differentiation (Lecca et al., 2016; Marangon et al., 2020).

OPCs (oligodendrocyte progenitor cells) isolated from the spinal cord of mice with EAE also express high amounts of miR-125a-2p and its inhibition resulted in accelerated myelin production (Marangon et al., 2020). Furthermore, miR-125a-2p overexpression studies showed its involvement in the regulation of genes necessary for gap junctions and other adhesion molecules that are indispensable for OPCs maturation, and can be associated with demyelination (Marangon et al., 2021).

The study of these miRNAs has clarified gene expression control mechanisms that can be used as biomarkers for the development of MS disease and possible therapeutic targets.

### 2.3. Mitochondrial dysfunction

Cellular metabolism depends on the proper functioning of mitochondria. At a structural level, mitochondria have several compartments to ensure their proper functioning, thus mitochondria are delimited by the external mitochondrial membrane, and inside it have an internal mitochondrial membrane where the oxidative phosphorylation machinery (OXPHOS) is mounted; this is where the greatest function entrusted to the mitochondria is carried out, which is the generation of adenosine triphosphate (ATP; Protasoni and Zeviani, 2021).

When mitochondria are under stress, morphological changes can appear and present vacuoles inside (Zuo et al., 2021). Normally the TDP-43 protein is associated with the inner mitochondrial membrane since it seems to regulate mitochondrial activities. Some binding sites of TDP-43 to the mitochondria are called M1, M3, and M5. In ALS, the presence of TDP-43 proteins with mutations (Berning and Walker, 2019) and in large amounts can result in mitochondrial damage, causing their death through the activation of autophagy (Buratti, 2015) and mitophagy (Gautam et al., 2019). This not only affects mitochondrial functioning but also the metabolism of the cell itself, causing its death (Altanbyek et al., 2016; Wang et al., 2016).

The damage made to the mitochondria by the presence of TDP-43 in ALS causes the membrane potential to fall (m∆ψ), the production of ATP to decrease (Wang et al., 2016), the consumption of oxygen also decreases, and consequently, the amount of Ca^2+^ and NAD^+^ to decrease (Gautam et al., 2022). Apoptosis is activated when the mitochondria have sustained such severe damage (Dafinca et al., 2021; Figure 1C; Table 1).

If the TDP-43 protein colocalizes with the mitochondria (Wang et al., 2016), complex I malfunction and its subsequent disintegration occurs due to the large amount of TDP-43. This may be the direct cause of changes in mitochondrial morphology and dynamics (Wang et al., 2019; Zuo et al., 2021). In ALS, not only apoptosis is activated but also the activation of necrosis induced by TDP-43 has also been observed (Dhuriya and Sharma, 2018).

Correct mitochondrial function is key to the proper functioning of neurons. In MS, dysfunction at the mitochondrial level in the production of ATP causes neuronal activity to decrease and this can cause neuronal degeneration. The stress induced by the generation of ROS in MS exacerbates neuroinflammation causing neuronal death (Barcelos et al., 2019; Blagov et al., 2022; Figure 2C; Table 1).

As the damage in MS disease progresses, defects at the mitochondrial level increase, such as the presence of mutations in mitochondrial DNA (mtDNA), this inevitably leads to a decrease in energy production, which leads to the functional loss of mitochondrial functions, by changes in the expression of mitochondrial genes (Barcelos et al., 2019; Blagov et al., 2022; Table 1).

### 2.4. Proteostasis dysregulation

Different factors can disrupt the cell’s homeostasis and destabilize the proteins in the endoplasmic reticulum (ER). ER is a vital component in maintaining internal balance through the unfolded protein response (UPR) during normal conditions (Li et al., 2022).

However, in the presence of activated UPR, the Integrated Stress Response (ISR) gets triggered, causing the activation of eukaryotic translation initiation factor 2A (eIF2a), which decreases the generation of new proteins. Physiological changes in neurons caused by the stress response alter the long-term synaptic plasticity of cholinergic interneurons (Rittiner et al., 2016), new research indicates that it’s possible to change neuronal excitability in other nuclei as well (Helseth et al., 2021). ER stress-induced eIF2a activation also drives the expression of a cascade of UPR-targeted genes responsible for protein folding, autophagy, and apoptosis, through the activation of transcription factor 4 (ATF4; Dafinca et al., 2021).

Evidence suggesting proteasome malfunction in ALS has been long established. While there is low toxicity with an increase in misfolded proteins in MNs, toxicity increases significantly when the proteasome is inhibited or ceases to function (Kitamura et al., 2014). In ALS, a decrease in the constituent components of the proteasome has been observed in MN cell cultures, and this augments disease progression (Cheroni et al., 2009).

Studies in mice have demonstrated that mimicking ALS symptoms is possible through the dysfunction of the ubiquitin-proteasome system in MNs (Tashiro et al., 2012). Consequently, the functional impairment of the proteasome has been linked to the accumulation of inclusions of the TDP-43 protein, together with poly-GA inclusions (Guo et al., 2018; Riemenschneider et al., 2022; Figure 1D).

In individuals with MS, antibodies targeting the proteasome have been identified in the cerebrospinal fluid (Mayo et al., 2002). This results in a reduction of the proteolytic activity of subunit 20 of the proteasome in both white and gray matter (Zheng and Bizzozero, 2011), leading to a deficiency in proteostasis and increased accumulation of misfolded proteins in the soma. This generates toxic effects that can cause apoptosis, such as in the case of the bassoon protein (Schattling et al., 2019). Conversely, activation of the proteasome can generate a neuronal protective effect by decreasing the accumulation of protein deposits (Schattling et al., 2019; Figure 2D).

### 2.5. Generation of stress granules

When the cell antioxidant defenses are compromised, SGs are formed. The production of free radicals and ROS in the cell causes the agglutination of various cellular components found in the cytosol, some of the structures found in these clusters are translation initiation factors, ribosomal subunits, mRNA, and RBPs (Protter and Parker, 2016). SGs act as a temporary barrier to stress and develop in response to physiological damage to cells.

The conglomerates that give rise to SGs are formed in the cytoplasm where they are completely assembled (Anderson and Kedersha, 2008; Harrison and Shorter, 2017). Proteins with mutations such as some RBPs, including the TDP-43 protein and the hnRNP A1 protein, can be sequestered by these protein conglomerates (Kim et al., 2013; Boeynaems et al., 2016); These SGs can be harmful to the cell when excessive amounts of RBPs are found (Harrison and Shorter, 2017). If these SGs clusters are removed, neurotoxicity can be decreased (Becker et al., 2017; Figure 1E; Table 1).

The lack of ROS clearance and the presence of the SOD1 mutant protein are the first signs of oxidative stress in ALS (Kirkinezos et al., 2005; Berdyński et al., 2022). When large conglomerates of SGs are made, it is when a proteinopathy that is mediated by the amount of TDP-43 occurs (Li et al., 2013; Wang et al., 2020).

Due to elevated ROS, TDP-43 protein clumps in ALS patients (Ratti et al., 2020; Zuo et al., 2021). Furthermore, SGs formation is induced (Ratti et al., 2020) due to the increased accumulation of TDP-43, this increase in SGs impairs nucleocytoplasmic transport, permitting the buildup of nucleocytoplasmic transport factors and RNA/protein complexes in the cytoplasm (Zhang et al., 2018; Chen and Cohen, 2019). TDP-43 mutations are one of the additional causes of SGs in ALS for example Q331K, A315T, or Q343R (Liu-Yesucevitz et al., 2010).

MS and EAE showed pathogenic RBP (hnRNP A1) dysfunction. This RBP rises up in the cytoplasm instead of the nucleus causing cytoplasmic mislocalization and showing accumulation in SGs (Douglas et al., 2016; Salapa et al., 2018). Also, dysfunction of this RBP increases the formation of SGs (Clarke et al., 2021). TDP-43 and hnRNP A1 can interact, and their colocalization in SGs structures further highlights the significance of SGs in MS (Libner et al., 2020; Salapa H. E. et al., 2020; Figure 2E; Table 1).

### 2.6. Hyperexcitability

The change in neuronal excitability is a consequence of different factors such as the increase in cellular stress and ROS by misfolding proteins. At different stages of ALS, patients have shown hyperexcitability in cortical MNs (Table 1; Vucic et al., 2008). These neurons from the patient cortex were used to have induced pluripotent stem cells (iPSCs) and these cells show *TARDBP* or *C9orf72* dysfunction, also their excitability response and synaptic plasticity were increased (Devlin et al., 2015; Perkins et al., 2021). The alterations observed might be attributed to the rise in calcium levels within the cell (Burley et al., 2022), or elevated glutamate receptor expression levels (Shi et al., 2019; Figure 1F).

This increase in calcium levels led to hyperexcitability and then to excitotoxic MN degeneration (Table 1; Ramírez-Jarquín and Tapia, 2018). Although it has been discussed whether the change in the expression of sodium channels on the surface of the MN is also involved, it is the change in the permeability of α-amino- 3-hydroxy- 5-methyl-4-isoxazole propionic acid (AMPA) (overactivation of AMPA receptors) or N-methyl-D-aspartate (NMDA) receptors that leads to Glutamate-mediated excitotoxicity (Netzahualcoyotzi and Tapia, 2015; Santa-Cruz et al., 2016).

These changes in excitability were reproduced in the SOD1^G93A^ animal model, showing less contribution of persistent γ-Aminobutyric acid (GABA) current in trigeminal MNs and an increase of L-Type Ca^2+^ currents. All these changes contributed to the imbalance in excitation-inhibition during the pathology (Laszlo et al., 2022; Venugopal et al., 2023).

There are several sources of excitability in MNs, astrocytes being one of them. In ALS, astrocyte-mediated neuronal hyperexcitability could happen via connexin 43 (Cx43) gap junctions (Almad et al., 2022). Loss of astrocyte Cx43 increases MN survival and decreases disease progression (LoRusso et al., 2019). An increase in Cx43 expression in astrocytes reduces MNs survival (Almad et al., 2016; Figure 1F).

Hyperexcitability in MS is closely related to the inflammatory state (Table 1). In patients, an increase in several cytokines, including interleukin 1 beta (IL-1β) and tumor necrosis factor (TNF), has been found in the cerebrospinal fluid. This increase is enough to raise the excitability of neurons in corticostriatal synapses (Rossi et al., 2012a, 2014). Also, IL-1β has a dual effect because it inhibits GABA transmission and reduces the spontaneous inhibitory postsynaptic currents (sIPSC; Rossi et al., 2012b), both effects contributing to possible excitotoxic neurodegeneration. In an animal model of MS, research findings suggest that the AMPA/kainate receptors are responsible for the mediation of glutamate excitotoxicity in OLs (Pitt et al., 2000; Figure 2F).

### 2.7. Axonopathies

Axonal degeneration, commonly referred to as “axonopathy” can be an early symptom of neurodegeneration. Thus, the first sign of axonal mitochondrial malfunction in response to oxidative stress and neuroinflammation is the dysregulation of mitochondrial transport (Brown and Bal-Price, 2003; Errea et al., 2015). This axon degeneration may be brought on by low ATP levels activating the protease and Ca^2+^ changes (Dutta et al., 2006) which could be specific by neuron subtype.

Recently, it was shown that myelin attenuates calcium transient currents via clusters of mitochondria, specifically on parvalbumin (PV) interneurons. The number of mitochondria is increased in glutamatergic neurons, whereas it is decreased in PV-GABA interneurons, which have lower overall excitability (Ramírez-Jarquín and Tapia, 2018; Kole et al., 2022).

Axonal transport (AT) directs the delivery of organelles from the cell body to synaptic compartments, removes and degrades unusable materials, and preserves the structural and metabolic variability of axonal subdomains (Brady and Morfini, 2017).

In healthy neurons, phosphorylation processes are precisely controlled both spatially and temporally, but these processes are frequently disrupted in ALS. Several of the kinase pathways linked to ALS produce an aberrant pattern of phosphorylation of numerous neuronal protein substrates that influences AT, which in turn promotes synaptic dysfunction and neuritic pathology in affected neurons (Guo et al., 2020). Thus, AT is a crucial cellular process that underlies the formation and maintenance of neuronal architecture and connectivity over the course of a neuron’s lengthy lifespan (Morfini et al., 2009; Brady and Morfini, 2017; Figure 1G).

It is well known that ALS is characterized by the activation of certain kinases, such as P38 MAP, in the MNs of the spinal cords of both sporadic and familial ALS patients as well as animal models (Bendotti et al., 2004; Sama et al., 2017). But also MAP/microtubule affinity-regulating kinases (MARKs), cyclin-dependent protein kinase 5 (cdk5), casein kinase 2 (CK2), and casein kinase I (CK1) are among the other kinases that assist with microtubule stability, within these kinases, glycogen synthase kinase 3 (GSK3) is the kinase activity that is most consistently linked to ALS patients, and its inactivation improves the survival of the neuron (Hu et al., 2003; Gibbs et al., 2018).

Multiple mechanisms can cause axonal damage in MS, which can occur at various stages of the disease. During the initial inflammatory phases, the primary cause of axonal damage is the inflammatory cascade, microglia activation, oxidative stress, and mitochondrial dysfunction. In contrast, during the later chronic stages, the loss of trophic support from myelin leads to an increase in energy demands. This results in the redistribution of sodium channels, an increase in calcium inside axons, and ultimately axonal fragmentation due to insufficient energy supply (Errea et al., 2015).

Astrocytes and/or microglia become “activated” by inflammatory mediators in MS. Microglia apparently release high levels of glutamate via a specific transporter in exchange for cystine, and this release is increased by inflammatory activation of the microglia. The reversible sodium-dependent glutamate transporter may also have increased activity in activated microglia and result in glutamate release in conditions where extracellular potassium is raised. It has been suggested that the mechanisms by which activated glia lead to neuronal death *in vitro* include the release of nitric oxide, ROS, glutamate and/or cytokines (Brown and Bal-Price, 2003; Figure 2G).

Activated microglia and macrophages are found in MS in close association with damaged axons. The expression of inducible nitric oxide synthase (iNOS) in astrocytes and/or the presence of nitrotyrosine were also found in chronic and acute MS lesions in postmortem tissues of MS patients. Nitric oxide (NO) could play a role in the pathology of demyelination and destruction of the OLs as this type of glial cell is also sensitive to NO induced cell death (Brown and Bal-Price, 2003).

In MS, an abnormal accumulation of glutamate in the synaptic cleft can occur due to an increase in release and/or a deficiency in the reuptake of glutamate by astrocytes. This can lead to overstimulation of glutamate receptors (GluRs) and cause excitotoxic damage to neurons and OLs. The downregulation of metabolizing enzymes, glutamate dehydrogenase, and glutamine synthetase in OLs exacerbates this effect.

In addition, the upregulation of glutaminase, an enzyme responsible for glutamate synthesis, is observed in MS lesions. This creates a permanent increase in proinflammatory cytokine levels, and the increased availability of glutamate can upregulate neuronal GluR expression, worsen synaptic dysfunction, and reinforce the local glutamate excitotoxicity (Sulkowski et al., 2014; Mandolesi et al., 2015).

In contrast to the excess of glutamate, GABA levels are reduced in both the CSF and CNS in patients with MS. Furthermore, the molecular mechanisms responsible for regulating GABA levels appear to be dysregulated in MS patients (Dutta et al., 2006).

Finally, neuroinflammation can affect synaptic transmission at different levels, with a huge impact on synaptic excitability and, therefore, neuronal function. Several characteristics of the spontaneous excitatory and inhibitory postsynaptic currents (sEPSCs and sIPSCs) recorded from neurons are affected by inflammation (Mandolesi et al., 2015).

## 3. Different features in pathophysiology

### 3.1. Lysosome dysfunction and autophagy dysregulation in ALS

ALS is closely linked with the hexanucleotide repeat expansion present in the *C9orf72* gene (Renton et al., 2011; Yang et al., 2020). The *C9orf72* gene is associated with proteins involved in autophagy initiation, Table 1 (Sullivan et al., 2016). Therefore, a malfunction in the *C9orf72* causes autophagy and lysosome defects that increase cell deterioration in ALS. During the early stages of the disease, autophagy can have beneficial effects, however, during the later stages, it can actually exacerbate the pathology (Rudnick et al., 2017).

Also, progressive lysosomal deficits in MNs impair autophagic degradation because autophagosome generation has limited responsiveness to proteotoxic stress (Xie et al., 2015; Evans and Holzbaur, 2019). Another mechanism by which there is a dysfunction in the regulation of lysosomes and autophagy is through TDP-43 which induces the nuclear translocalization of Transcription Factor EB (TFEB), which regulates autophagy and lysosome biogenesis (Xia et al., 2016; Figure 1H; Table 1). In ALS-like models, it was found that RBPs accumulation impairs autophagy via mTOR dysregulation (Paul et al., 2023).

### 3.2. Inflammation in ALS

#### 3.2.1. Innate immune activation

At the beginning of the ALS disease, it has been reported that there is a mislocalization and the presence of TDP-43 protein aggregates not only in the cytoplasm of neurons but also in the glia of the CNS (Figures 1A,B). These situations can activate the signaling pathway mediated by the NF-κβ, a transcription factor responsible for controlling the activation of various proinflammatory pathways (Liu T. et al., 2017). Some of the proinflammatory cytokines that are activated by this factor in ALS are IL-6, TNF-α, cyclooxygenase 2 (COX-2), and prostaglandin E-2 (PGE-2; Moreau et al., 2010).

Another signaling pathway involved in the activation of the immune response due to the presence of TDP-43 aggregates in ALS is that of the inflammasome (Table 1) a member of the NOD-like receptors (NLRP3). The stimulation of NLRP3 causes overexpression of the proinflammatory cytokine IL-1β (Bright et al., 2021); can inhibit the cytosolic Parkin E3 ubiquitin ligase, responsible for the degradation of protein aggregates such as RBPs, generating neurotoxicity by the presence of TDP-43 in neurons (Zhuang et al., 2017) and also can generate cellular stress and cause neurodegeneration by stimulating an imbalance at the cellular level, which can damage the mitochondria and prolong the activation of the immune response (Sliter et al., 2018).

Activation of the NF-κβ and NLRP3 pathways results in the production of cytokines such as interleukin 1β (IL-1β; Hu et al., 2017) and IL-6, which have pro-inflammatory and anti-inflammatory functions, respectively, (Pronto-Laborinho et al., 2019; Figure 1I). Other elevated interleukins are IL-13 (Lu et al., 2016) and IL-18 (Italiani et al., 2014).

According to Lee et al. (2018), there is an increase in the expression of the complement pathway in ALS (C5a and the C5a receptor), which facilitates the loss of communication at the neuromuscular junction and neuronal death. This complement activation activates the production of TNF, another proinflammatory cytokine (Guidotti et al., 2021).

The activation of the immune system and its production of NFκβ or TNF-α either through the NLRP3 pathway or the complement pathway, respectively, leads to elevated total peripheral blood leukocyte count in ALS (Murdock et al., 2016). In addition, there is an increase in the total number of granulocytes and the presence of neutrophils and monocytes (Murdock et al., 2016).

The neuroinflammation may also be mediated at the genetic level by abnormalities in the *C9orf72* gene (Yang et al., 2020). The presence of this gene induces the overexpression of IL-6 and IL-1β in microglia. These cytokines condition inflammation at the peripheral level and at the CNS level (Bright et al., 2019).

The CNS cells that express the *C9orf72* gene are dendritic cells (DCs) and microglia (O'Rourke et al., 2016). Abnormalities in the *C9orf72* gene may render microglia unable to disintegrate aggregated proteins such as RBPs, which may lead to chronic activation of microglia by stimulating the expression of proinflammatory cytokines that promote neuroinflammation (O'Rourke et al., 2016).

#### 3.2.2. Adaptive immune activation

As mentioned, the immune system may be involved in the prolonged maintenance of neuroinflammation in ALS, which can cause the death of MNs in the CNS and spinal cord, however, the immune mechanisms that cause this damage are not fully understood. The precise contribution of adaptive immunity in the pathogenesis of ALS is a recent area of investigation.

The presence and mislocalization of TDP-43 and its aggregates in the cytosol can activate the innate immune response in a prolonged and sustained manner, which causes neuroinflammation and leading the overproduction of proinflammatory cytokines such as NF-κβ, IL-1β (Table 1), IL-6, TNF-α (Table 1), IL-13, and IL-18 which can chronically activate microglia in the CNS, allowing T-lymphocyte infiltration.

These T cells directly interact with microglia in CNS and chronically promote neuroinflammation that ultimately leads to irreversible neuronal damage. Microglia and macrophages participate in axonal regeneration, but as there is chronic overexpression of proinflammatory cytokines, this activity is decreased, which contributes to neurodegeneration. Neuroinflammation chronically produces interferon gamma (IFNγ) and causes high infiltration of T lymphocytes into the CNS for some as yet unknown reason (Beers et al., 2017).

In ALS the population of regulatory T lymphocytes (Tregs) is decreased. This condition exacerbates the neuroinflammatory process, leading to accelerated MNs death and shortening of survival (Beers et al., 2017).

One of the main functions of these Treg lymphocytes is to decrease the production of TNF-α and IFNγ and highly proinflammatory cytokines; In ALS, if the activity of these Treg lymphocytes were correct, the intensity of the inflammation would be reduced, thus reducing the damage (Beers et al., 2017). But on the contrary, its low presence in ALS means that the immune response does not have control, and this leads to irreversible damage.

The severity and poor prognosis of ALS are largely due to the abrupt and acute activation of the immune response, that generates a greater amount of irreversible damage (Holmoy, 2008).

### 3.3. Immune response in MS

An essential feature of MS is the presence of demyelinating plaques present in the CNS and the spinal cord, leading to long-term axonal loss; these damages are due to chronic inflammation (Monaghan and Wan, 2020).

MS also has features of an autoimmune disorder, as there is the abnormal presence of T helper 1 (Th-1) and Th-17 lymphocytes in the CNS, these lymphocytes secrete proinflammatory cytokines which continue to chronically damage the entire CNS. This chronic neuroinflammation will determine the progression of the disease (Mcginley et al., 2018; Nitsch et al., 2021).

The presence of antibodies to attack myelin causes the loss of the protective covering of the nerve fibers of the neurons, causing deficiencies in the transmission of the nerve impulse by losing its protection (Ransohoff, 2018; Nitsch et al., 2021; Figure 2H).

#### 3.3.1. Innate immune activation

PRRs on cells of the innate immune system (Mcginley et al., 2018) are activated by binding to PAMP (from pathogens or commensal bacteria) and DAMPs (dead or inactive cells). In the particular case of MS, the innate immune system recognizes the TDP-43 protein as a DAMP, Table 1 (Mcginley et al., 2018).

This first recognition triggers initial damage that will result in the production of proinflammatory cytokines such as IL-1, IL-6, IL-12, IL-18, and IL-23, activating Th1, and Th17 lymphocytes (Table 1) and activating the cells of the adaptive system such as T and B lymphocytes (Mcginley et al., 2018; Figure 2I).

Once the innate immune response is on, the displacement of leukocytes toward the brain parenchyma causes the activation of mast cells. With this, the integrity of the BBB is compromised, which causes the free passage toward the CNS of proinflammatory cytokines that alter the cellular environment (Lopes Pinheiro et al., 2016).

#### 3.3.2. Adaptive immune activation

The adequate maintenance of the cellular immune response is possible thanks to the activation of regulatory T lymphocytes (CD4^+^) and cytotoxic T lymphocytes (CD8^+^). Inherently, activation of the Foxp3 transcription factor aids the proper function of these lymphocytes by allowing the maintenance of immune tolerance by regulating immune cell activation.

If Foxp3 is removed from CD4^+^ lymphocytes, autoimmunity problems arise, which are the main feature of neurodegeneration in MS. However, if Foxp3 expression on CD4^+^ lymphocytes is normal, then CD8^+^ T lymphocytes may be inhibited (Josefowicz et al., 2012).

Although during the development of neuroinflammation in MS there are CD4^+^ (Treg) lymphocytes in the CNS, these are found in fewer numbers and the few that do exist have a diminished capacity to control the inflammatory activity of Th-1 and Th-17 lymphocytes (Table 1; Josefowicz et al., 2012; Mcginley et al., 2018).

As mentioned above, the firing of the innate immune response leads to the expression of IL-1. This activation triggers a series of signaling cascades in activated T cells, allowing the expression of other interleukins such as IL-17, IL-21, and IL-23. These interleukins, particularly IL-17, activate the Th-17 response in these T lymphocytes, promoting the amplification of immune signaling (Mcginley et al., 2018).

This inflammation can begin at the peripheral level, causing the BBB to become permeable, allowing activated T cells (Th1 and Th17) to migrate and continue to express other proinflammatory cytokines such as IL-1 (Table 1) in neutrophils, monocytes, and microglia at the CNS level (Sospedra and Martin, 2016; Mcginley et al., 2018).

Other proinflammatory cytokine that is activated include the granulocyte-macrophage stimulator (GM-CSF) which activates microglia and induces the expression of IFN𝛄 and TNF𝛂 (Table 1) causing neuronal damage due to neuroinflammation stimulated by neurotoxicity (Mcginley et al., 2018).

The initiation and perpetuation of this neuroinflammation cause axonal damage by destroying the myelin sheaths, at which point the clinical signs and symptoms of MS become visible (Mcginley et al., 2018; Álvarez-Sànchez et al., 2019).

At the same time, there is also an exacerbated stimulation of phagocytes in the CNS and these may be responsible for the damage to the myelin sheaths of the axon too (Prineas and Parratt, 2021).

Other cells involved in the development of the autoimmunity process in MS are B lymphocytes whose main activity is the production of antibodies and in the particular case of MS, these antibodies are directed specifically against the myelin sheath (Table 1; Comi et al., 2021).

These antibodies against the myelin circulating in the CNS, by leaving the neuronal axon bare, cause its destruction. This demyelination mediated by neuroinflammatory processes initiates the autoimmune process by generating autoantibodies against myelin through the activation of the B lymphocyte (Table 1; Josefowicz et al., 2012).

Another aberrant role mediated by B lymphocytes in MS is to stimulate the expression of pro-inflammatory cytokines such as GM-CSF and IL-6 with a decrease in anti-inflammatory cytokines such as IL-10 (Monaghan and Wan, 2020; Figure 2I).

There are reports showing the presence of antibodies not only against myelin but also against hnRNP A1 (Yukitake et al., 2008; Lee et al., 2011) in the neuronal bodies of EAE mice (Kuerten et al., 2020; Libner et al., 2020, 2022). These antibodies against the hnRNP A1 protein are found in the periphery around neurons in the spinal cord. It seems that this RBP is intact and is found inside neurons, but by still unknown mechanisms it can be fragmented and these pieces of protein can be exocytosed from the neuron and at some point give rise to anti-hnRNP A1, causing neuronal damage (Libner et al., 2020).

A risk factor that has been associated with the development of MS disease is the presence of allelic variants (DRB1*1501, *0301, and *1303) of the protein called human leukocyte antigen (HLA class II) (International Multiple Sclerosis Genetics Consortium et al., 2011; Ransohoff, 2018). These allelic variants seem to have the ability to bind to myelin and recognize it as an antigen and that is when neurons can suffer neurodegeneration as they are a direct target in their axonal portion where the myelin sheath is found, exacerbating the autoimmunity initiated by B lymphocyte (Ransohoff, 2018).

Thus, these aforementioned allelic variants are associated with an increased risk not only of developing MS but also involved in the maintenance of said autoimmunity (Ransohoff, 2018).

### 3.4. Neurodegeneration

In ALS, cells are not equally affected by degeneration, even though proteins like TDP-43 or mutant SOD1 are expressed ubiquitously, for example, abnormal TDP-43 can be found in the basal ganglia and the substantia nigra, those areas are not affected in the disease. A wealth of evidence shows that some MNs subpopulations are more vulnerable than others to cell damage but it is unknown why. Spinal and hypoglossal MNs are among the first to degenerate and, oculomotor neurons and Onuf’s nuclei MNs are last to do so, this is why vision, sexual, and bladder function are maintained undisrupted during the course of the disease (Ragagnin et al., 2019).

Extrinsic factors like the type and density of non-neuronal cells or GABA input from interneurons, and intrinsic factors like neural excitability and receptors for inflammatory molecules, determine the susceptibility to neurodegeneration by excitotoxicity in ALS (Nijssen et al., 2017; Ragagnin et al., 2019; Allodi et al., 2021), such as the fast-twitch motor neuron alpha motor neuron (FF-alfa-MN), which primarily degenerates before the cranial nerves III, IV, and VI that control motor ocular movements (Caligari et al., 2013).

However, using the connectome, which indicates trans-synaptic pathways where interneurons are important, the propagation could be accomplished through either continuous or discontinuous spread (Hossaini et al., 2011; Kanouchi et al., 2012; de Carvalho et al., 2014) as in work by You et al. (2022) where the PV positive interneurons and alpha MNs are lost in the model of ALS.

Neurodegeneration is a component of MS, present in all stages of the pathology, even in RRMS (Mey et al., 2023). For many years, neurodegeneration was thought to be a consequence of inflammatory demyelination, but some research suggests that neurodegeneration occurs in all disease stages (Trapp and Nave, 2008; Frischer et al., 2009; Lassmann and van Horssen, 2011). Biopsies from patients with MS show an association between demyelinated plaques and axonal loss in the white matter tracts of the spinal cord (Table 1), suggesting that their mechanism probably contributes to neurodegeneration in the disease (Dutta et al., 2006; Levin et al., 2014; Mandolesi et al., 2015).

MS progression does not seem to follow any pattern like ALS does, that is, there is no susceptibility to damage depending on cellular subtypes. Actually, MS lesions are disseminated in space and time and may include IgG oligoclonal bands in the cerebrospinal fluid (Thompson et al., 2018). Patients may or may not experience inflammatory disease activity and the disease can follow various courses, relapsing–remitting MS being the most common one (Confavreux et al., 2020; Mey et al., 2023).

During the early stages of MS, the brain attempts to compensate for demyelination by redistributing voltage-gated Na^+^ channels that were concentrated in the Ranvier nodes, along the axon; this allows for slow but effective nerve conduction (Dutta et al., 2006). Senol et al. (2022) show that there are alterations in the initial segment of axons that could possibly lead to increased neural excitability, in both active and inactive lesions from patients.

Mitochondrial dysfunction and reorganization of voltage-gated Na^+^ channels might be one of the main drivers of degeneration in advanced stages of MS. Evidence shows that upper MNs firing rates increase, in part due to a lack of inhibition, given that GABAergic neurotransmission is reduced pre and postsynaptically in MS patients. This translates into a higher energetic demand that cannot be met due to mitochondrial damage, as described above.

This increases Na^+^ levels inside the cell, that under non-pathological conditions would be exchanged for extracellular K^+^ by the Na^+^/K^+^ ATPase. It is believed that intracellular Na^+^ can be exchanged by the Na^+^/Ca^2+^ exchanger that is non-ATP dependent, increasing axoplasmic Ca^2+^ and resulting in degeneration (Figure 2F; Dutta et al., 2006). In postmortem studies, neurons from patients with progressive MS show severe mitochondrial dysfunction, given the decreased activity of complexes I and III from the respiratory chain (Nikić et al., 2011; Libner et al., 2020).

### 3.5. Alterations in the periphery

Accumulations of phosphorylated Ser409/410 TDP-43 (pTDP-43) were found in intramuscular nerve bundles (INBs) in postmortem biopsies from patients with sporadic ALS (sALS). Also, a study in 114 biopsies from patients with no muscle diseases and/or ALS causing variants was done; patients with abnormal deposits of pTDP-43 in INBs were later diagnosed with ALS, suggesting that these findings could lead to a new diagnostic biomarker for the disease (Kurashige et al., 2022).

Even though TDP-43 accumulation has been reported in MS and EAE, there are currently no studies that show its dysfunctionality in muscular structures.

Another important feature of ALS is the disruption of the neuromuscular junction (NMJ; Bruneteau et al., 2013; Badu-Mensah et al., 2022). To this day, there are no reports of how MS pathology affects the structure of the neuromuscular junction (NMJ).

#### 3.5.1. Oligodendrocytes, oligodendrocyte progenitor cells, and myelin in ALS and MS

OlLs in the ALS pathology were not considered to play a relevant part in de onset and/or development of the disease until 2013 when TDP-43-positive aggregates in neurons and OLs in spinal cord samples from patients were reported; they also described abundant degenerative changes in OLs (Table 1; Philips et al., 2013; Pons et al., 2020).

Similar findings were reported for the SOD1^G93A^ mouse. In animals they detected morphological changes in the gray matter before disease onset: OLs in the ventral gray matter had a thicker cell body and a more elongated morphology, and these abnormal cells increased in number as the pathology progressed, eventually resulting in apoptotic OLs.

OLs numbers were maintained along the disease because of compensatory mechanisms: nerve/glial antigen 2 (NG2) OLs/glia precursors increase their rate of proliferation and differentiation in response to the loss of OLs, nevertheless these cells showed reduced myelin basic protein and monocarboxylate transporter 1 (MCT1), which translates to impaired myelination and metabolic support, suggesting this could contribute to MNs death.

These results, backed up by multiple other articles, highlight non-neuronal cells as a possible target not only for the treatment of ALS but for the expansion of basic knowledge of pathology (Philips et al., 2013; Pons et al., 2020; Heo et al., 2022).

Even though RBPs dysfunction has been found in OLs in both pathologies (Lu et al., 2016; Masaki et al., 2020; Jahan-Abad et al., 2023), cells are affected very differently; in MS, active demyelinating lesions (Figure 2J) suggest the preservation of OLs because numbers are comparable to the ones in normal-appearing white matter, on the other hand, chronic lesions show significant cell loss (Prineas and Parratt, 2012; Heß et al., 2020). In acute lesions, OLs retract their processes from axons but their somas remain intact, suggesting a stage of sub-lethal injury (Molina-Gonzalez et al., 2022).

The physiological presence of TDP-43 is essential for the survival and myelination of the OL, this physiological function does not cause neuronal death or denervation of the NMJ (Wang et al., 2018; Heo et al., 2022).

## 4. Experimental models for ALS and MS

### 4.1. ALS animal models

The generation of animal models for the study of ALS can be difficult, due to the fact that there are two main variants of the disease; sporadic and familial. There are no models for sALS but great progress has been made with the ones that replicate a known mutation from familial ALS (fALS).

The first ever transgenic model for ALS was created by Gurney and colleagues in 1994 by expressing a mutant form of the human *SOD* gene, this produced paralyzed mice with MN loss from the spinal cord and eventually death. Other transgenic models, targeting different genes have been elaborated, for example, *C9orf72* knockdown zebrafish model (Ciura et al., 2013), FVB-*C9orf72* bacterial artificial chromosome (BAC) mouse model (Liu et al., 2016), TDP43-Q331K mouse model (Arnold et al., 2013).

Given the importance of TDP-43 aggregation in the pathology, several models were developed, being ALS10 the first one; in 2009 a mouse expressing the disease-associated mutant TDP43-A315T was created (Table 1; Wegorzewska et al., 2009). More modern models express TDP-43 at more endogenous levels, like the single-copy BAC model of TDP43-M337V (Todd and Petrucelli, 2022).

Another approach is to focus on one hallmark of the pathology, and to try to reproduce it in animals like the group of Ricardo Tapia did, by developing a model of MN death induced by excitotoxicity; In this model, a glutamate receptor agonist, AMPA, is administered with a mini osmotic pump (Almoshari, 2022) directly into the ventral horns of the spinal cord, resulting in progressive paralysis of lower limbs and death of MNs (Tovar-Y-Romo et al., 2007).

*In vitro* models such as ALS-on-a-chip technology can implement optogenetics for the induction of MNs spheroids (Osaki et al., 2018) or TDP-43 opto-aggregation can be induced in cell cultures (Mann et al., 2019).

OptoGranueles is another approach that uses optogenetics for the multimerization of some scaffold proteins necessary for SD assembly (Zhang et al., 2019).

Lastly, in 2020, Asakawa et al. (2020) developed an *in vivo* model that uses optogenetic TDP-43 (opTDP-43) in zebrafish. Light stimulation induced cytoplasmic opTDP-43 mislocalization in spinal MNs.

### 4.2. EAE animal models

MS is a very complex and heterogeneous pathology, with an unknown origin that can affect patients in several ways, thus its study in the laboratory becomes challenging.

There are many animal models that have been developed with the intention to mimic MS as closely as possible and even though models are imperfect, great advances have been made. EAE or experimental autoimmune encephalomyelitis was developed by Rivers in 1933 and it is the most commonly used animal model in MS research (Constantinescu et al., 2011).

Nowadays EAE is induced by administering animals with immune triggering peptides such as myelin OLs glycoprotein (MOG), myelin proteolipid protein (PLP) and Myelin basic protein (MBP) emulsified in Freund’s complete adjuvant (CFA) (Laaker et al., 2021; Huntemann et al., 2022; Takano et al., 2023) and complementing with Pertussis toxin (PTx) to permeate the BBB and increase disease severity (Singh et al., 2021; Zuo et al., 2022).

The type of immune response that will be developed depends on the animal strain and the protein used, among other factors. In C57BL/6 mice, MOG_35-55_ results in a T-cell mediated response (Saligrama et al., 2019; Huntemann et al., 2022) whereas, MOG_1-125_ produces a B-cell dependent outcome (Stevens et al., 2020). Another popular alternative is the SJL mouse, which responds better to PLP induction and is useful for the study of EAE’s initial wave.

A great number of authors report a higher susceptibility to EAE in females (Rassy et al., 2020) but, some articles report results in mixed genre groups, making no distinction in sex susceptibility (Othy et al., 2020).

EAE is a useful model to understand the pathogenesis of MS, however, there are several differences; in the EAE model, greater damage is seen in the white matter of the spinal cord, while in patients with MS, there is demyelination mainly in the cortex and cerebellum. It seems that the neuroinflammation is of a different origin in the EAE, causing this damage in the spinal cord and not in the cortex and cerebellum as would be expected in MS (Kutzelnigg et al., 2007; Mcginley et al., 2018).

Viral-induced models are another option for the study of such pathology being Theiler’s murine encephalomyelitis virus (TMEV) (DePaula-Silva et al., 2017) and the murine hepatitis virus (MHV) the most common ones. Since some of the viral induced models can cause more severe symptoms than EAE models do, they are harder to work with.

Some of the viruses can also work as seizure generating agents (Löscher and Howe, 2022), making them useful models for epilepsy (Patel et al., 2022). These models can help study mechanisms for myelin loss that might not be immune-mediated by B and/or T cells (Buttigieg et al., 2023).

Lastly, demyelination in mammals can be triggered by toxic reagents such as ethidium bromide (EtBr), lysolecithin (LPC), and cuprizone. Some of them can be selective to a cell type, by various mechanisms, for example, cuprizone induces OLs apoptosis by homeostasis alteration and, lysolecithin produces OLs death by dissolving its cell membrane given its detergent properties. EtBr is non cell specific (Buttigieg et al., 2023).

## 5. Discussion

We now recognize the interconnected relationship between inflammation and neurodegeneration as a result of advancements in our understanding of how protein aggregation by RBPs induces damage and activates the immune response in both ALS and MS. In Table 1, we provide a comparative summary of characteristics in ALS, MS, EAE, and ALS models at a molecular level.

Selective activation of the immune system provides further evidence of the dysregulation in the immune system in ALS, in this pathology immunity is altered by the presence of TDP-43 aggregates, however, more research is needed in order to establish the mechanisms through which TDP-43 selectively activates the innate immune system.

On other hand the activation of the adaptive immune response in MS is responsible for the damage caused in the disease, but there is also presence of RBPs thus, there are unresolved questions about some features of the disease such as: Why are RBP autoantibodies present in both diseases? (Yukitake et al., 2008; Lee et al., 2011; Conti et al., 2021; Nielsen et al., 2021).

Neurodegeneration is present in MS along with a marked immunological response against myelin, and the role of RBPs is beginning to be studied. RBP aggregation and neuroinflammation are both clearly visible in ALS. These points of intersection in the process and development of diseases (ALS and MS) could be the beginning of new investigations where the interrelationship between the dysfunction of the immune response and the dysregulation of the CNS can be addressed.

**Figure 3:**
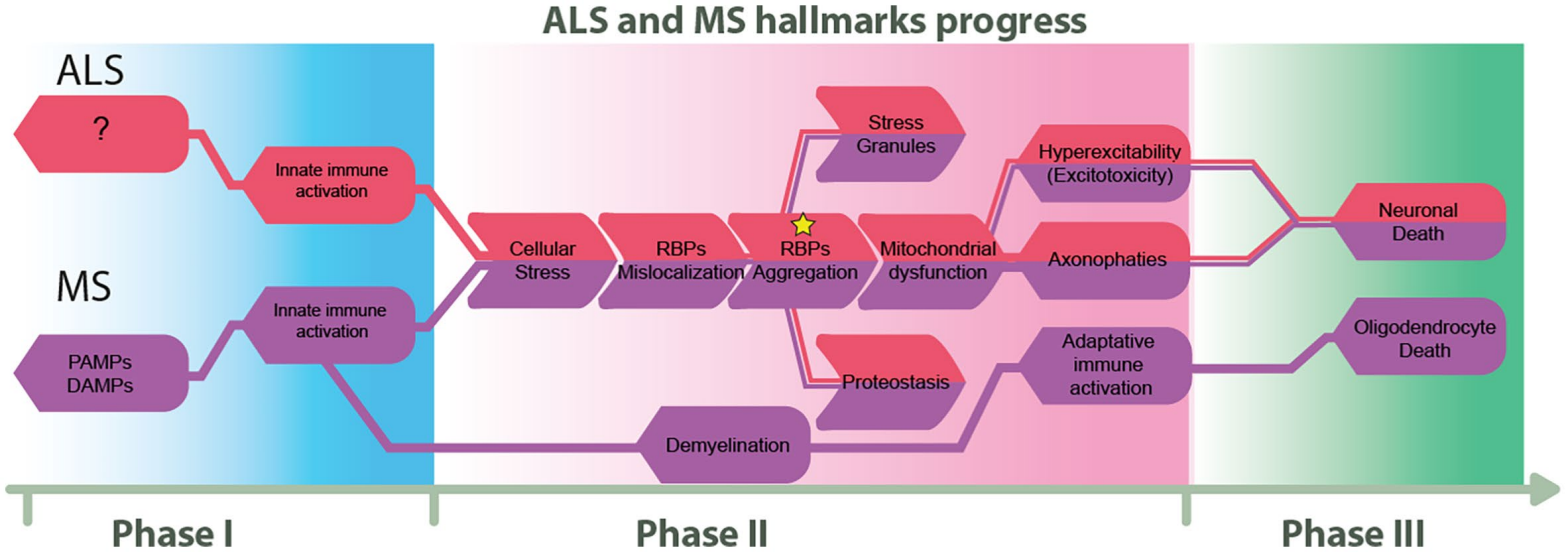
Proposed flow chart on the progress of ALS and MS. We describe three progressive phases that highlight the similarities and differences between ALS and MS. The first phase illustrates the principal known triggers of these diseases. The second phase highlights shared molecular hallmarks, and finally, the third phase represents imminent cellular death.

We propose in Figure 3 how ALS and MS seem to diverge at their onset and end, but there is a point in the disease course where some hallmarks are shared. This figure also proposes three phases of pathological progression, at the molecular level.

Maybe an efficient immune response is necessary to control the progression of ALS and, a regulation of neuroinflammation and neurodegeneration in MS will help to limit progression even in PPMS.

Initially, only MNs in ALS were known to present abnormal TDP-43 deposits, but recently, pathological aggregation of TDP-43 has been described in other cell types too, OLs being one of them; RBPs aggregation, as mentioned before, is a feature found in both ALS and MS OLs but, for some reason, the fate of these cells is very different in each pathology.

In acute and active lesions in MS, mature OLs seem to follow a series of events where their normal activity is disrupted but cells continue to survive. An obvious question arises; why during acute and active lesions, OLs are not able to effectively elongate their processes to cover axons?

It is known that metabolic stress, glutamate and inflammatory mediators play a central role here (Philips et al., 2013; Masaki et al., 2020; Molina-Gonzalez et al., 2022; Jahan-Abad et al., 2023) and since some evidence suggests that axonal damage may be present before demyelination (Dutta and Trapp, 2007; Simkins et al., 2021), neurons may be affected first but this is yet to be probed.

Another issue worth mentioning is the fact that abnormal protein aggregation is not spontaneous and there are several events that lead to an aggregate, each with consequences for the cell; to begin with, RBPs must be mislocalized out of the cellular nucleus. Interestingly normal functioning TDP-43 is a requirement for OLs survival and myelination but this has no effect in MNs and the NMJ (Wang et al., 2018).

In ALS, TDP-43 deposits are also present, OLs death is observed along the course of the disease, combined with high rates of defective OPCs proliferation (Philips et al., 2013), and since the simple removal of the TDP-43 protein from the nucleus of OLs is not enough to cause damage to the MN and NMJ, which is a necessary feature in ALS, additional abnormal processes must be involved. There is evidence for the existence of inclusions of the phosphorylated TDP-43 protein in regions of the intramuscular nerve bundles just before the onset of ALS (Kurashige et al., 2022), highlighting the role of events happening early in the periphery.

Why OLs and OPCs behave differently in MS and ALS even though RBPs aggregates are reported for both pathologies, remains an open question, but again, other cells may be decisive effectors in the divergence of ALS and MS pathophysiology.

Glial cells like astrocytes and microglia may be key players for both conditions: In the case of astrocytes, cross-talk between them and OLs is described via gap junctions made up of CX30 and CX46. In ALS astrocyte-mediated hyperexcitability happens through CX43. But there are also different ideas about the origin of the hyperexcitability of MNs in ALS.

On one hand, several studies have shown that there is an excess of glutamate in the synaptic space and that there is an overactivation of NMDA and AMPA receptors. This, in turn, can elevate intracellular sodium and calcium (Netzahualcoyotzi and Tapia, 2015; Ramírez-Jarquín and Tapia, 2018) causing an increase in cellular stress that can lead to the activation of cell death pathways.

Some models suggest that the overactivation of AMPA receptors is enough to observe neuronal deterioration. However, it has also been described that there are no changes in the number of receptors but in their conductivity (Rao and Balachandran, 2002; Van Damme et al., 2007). Additionally, there are a couple of experiments that mention an increase in glutamate receptors on the surface of the neuron. On the other hand, the inhibition of both the glycinergic and GABAergic pathways plays an important role in these ALS and MS. Thus, there is excitotoxicity in both conditions.

It decreases the expression of these receptors as well as the amount of their RNA transcripts, causing irreversible damage. Although these pieces of evidence may not necessarily be contradictory, multifactorial changes have been observed, and more than one change may coexist at the same time.

This review shows that RBPs like TDP-43 are present in ALS and MS, i.e., they are a common ground where both diseases stand, and that RBPs are involved in other of the pathological hallmarks described here. The integration of this information may change the way we study and treat diseases. A more well-rounded approach is needed since neurodegeneration and inflammation are not separated and exclusive events for both ALS and MS but quite the opposite; they seem to be intermingled and degeneration can occur in all cells of the nervous system.

## Author contributions

IA-G, RH-M, and TR-C made equal contributions to research, writing and edition. RH-M made the figures. EC contributed to the writing, edition and acted as clinical advisor. JJA-B made conceptualization, research, writing, and edition. All authors contributed to the article and approved the submitted version

## Conflict of interest

The authors declare that the research was conducted in the absence of any commercial or financial relationships that could be construed as a potential conflict of interest.

## Publisher’s note

All claims expressed in this article are solely those of the authors and do not necessarily represent those of their affiliated organizations, or those of the publisher, the editors and the reviewers. Any product that may be evaluated in this article, or claim that may be made by its manufacturer, is not guaranteed or endorsed by the publisher.
